# Biocontrol of Cereal Crop Diseases Using Streptomycetes

**DOI:** 10.3390/pathogens8020078

**Published:** 2019-06-13

**Authors:** Jake T. Newitt, Samuel M. M. Prudence, Matthew I. Hutchings, Sarah F. Worsley

**Affiliations:** School of Biological Sciences, University of East Anglia, Norwich Research Park, Norwich, Norfolk NR4 7TJ, UK; j.newitt@uea.ac.uk (J.T.N.); s.prudence@uea.ac.uk (S.M.M.P.)

**Keywords:** *Streptomyces*, biocontrol, cereals, root microbiome, rhizosphere

## Abstract

A growing world population and an increasing demand for greater food production requires that crop losses caused by pests and diseases are dramatically reduced. Concurrently, sustainability targets mean that alternatives to chemical pesticides are becoming increasingly desirable. Bacteria in the plant root microbiome can protect their plant host against pests and pathogenic infection. In particular, *Streptomyces* species are well-known to produce a range of secondary metabolites that can inhibit the growth of phytopathogens. *Streptomyces* are abundant in soils and are also enriched in the root microbiomes of many different plant species, including those grown as economically and nutritionally valuable cereal crops. In this review we discuss the potential of *Streptomyces* to protect against some of the most damaging cereal crop diseases, particularly those caused by fungal pathogens. We also explore factors that may improve the efficacy of these strains as biocontrol agents in situ, as well as the possibility of exploiting plant mechanisms, such as root exudation, that enable the recruitment of microbial species from the soil to the root microbiome. We argue that a greater understanding of these mechanisms may enable the development of protective plant root microbiomes with a greater abundance of beneficial bacteria, such as *Streptomyces* species.

## 1. Introduction

Cereal crops, or “cereals”, are plants belonging to the grass family *Poaceae* that are grown and harvested primarily for their edible grain [1]. The economic and social importance of cereal crops cannot be understated, as they provide fundamental nutrition for the vast majority of the world’s population. Most cereal crops are grown primarily for their grain, which contains a nutritional starchy endosperm, and forms a staple part of the human diet [1]. However, many cereals can also be used for the upkeep of animal livestock and their utility is further enhanced by their capacity for long term storage [1]. The focus of this review is directed at key global cereal crops, for example maize, wheat, rice, barley, sorghum, millet, oats, and rye [1]. The Food and Agriculture Organization (FAO) of the United Nations estimates that 2609 million tonnes of such cereal crops were produced in 2018 [2].

One of the greatest challenges facing the world today is to match the demand of a rapidly expanding global population with an increase in food production, whilst simultaneously ensuring that this is done sustainably and within the limitations of land availability for agriculture [3]. In order to meet this target, it will be necessary to pursue two intimately linked goals. The first is to increase crop yield, particularly that of cereal crops, which can be attained through various methods, such as selective breeding, genetic modification, as well as carefully controlled irrigation and fertilisation regimes [3,4]. The second is to minimise crop losses caused by pests and diseases, which are conservatively estimated to cause between 20–40% of losses to yield, with further consequences for livelihoods, public health and the environment [3,4,5,6,7]. The implementation of strategies to achieve the latter are challenging, particularly as the factors that underpin plant disease are highly complex and multivariate [6].

Many different types of organism can infect cereal crops, including a range of bacteria, oomycetes, fungi, viruses and nematodes [8]. Fungal diseases, in particular, are considered to be one of the most dominant groups of cereal crop pathogens, with agents causing disease at every level of plant physiology [8,9]. Different fungal infections can, thus, cause a wide range of symptoms that can all contribute to yield losses. For example, infection by several fungal pathogens results in the formation of necrotic lesions on leaves and stems that can eventually lead to leaf senescence and a reduction in grain quantity; this is the case for rust infections caused by *Puccinia* species and also for rice blast fungus, caused by the species *Magnoporthe oryzae* [8,9,10,11]. Rice blast can be incredibly destructive, and is estimated to be responsible for 30% of losses to rice crops globally [12]. Other pathogenic soil-borne fungal species invade primarily at the plant roots, causing root rot from the base of the plant upwards, whilst simultaneously sapping the host plant of its nutrients; this is the case for the causative agent of wheat Take-all disease, *Gaeumannomyces graminis*, which in some cases can eliminate an entire wheat crop [13]. Thus, *G. graminis* is often cited as the most important root disease of wheat worldwide [13,14,15,16]. Additionally, many fungal species (such as *Fusarium* spp.) do not cause plant senescence, but instead can negatively impact yield by causing a dramatic reduction in grain quality via the production of high concentrations of mycotoxins [8,17].

The most widely used method to combat the losses caused by crop disease is the routine application of chemical pesticides to crops, with the aim of eliminating or limiting the severity of disease phenotypes. However, it is increasingly becoming clear that the long-term use of chemical pesticides can have several negative side-effects. For example, many pesticides can lead to both acute and chronic toxicity in humans and they are increasingly being shown to cause wide-spread damage to the wider ecosystem by impacting non-target organisms, such as pollinator species, and also through the pollution of soil and water systems [18,19,20]. These non-target effects can also extend to reducing the diversity of beneficial microbial species in the soil, which can in turn release pathogen populations from competition and increase the chances of pathogen invasion [21]. The use of chemical pesticides is additionally hampered by the evolution of microbial resistance. In much the same way that we face a crisis in modern medicine due to antimicrobial resistance, so too do we face a decline in the effectiveness of pesticides due to phytopathogen resistance [22,23].

As a result of the issues and side-effects of using chemical pesticides to control crop diseases, research is beginning to re-focus on finding alternative solutions to combat pathogenic infection. Crop rotation has played a vital role in phytosanitation throughout history, and aims to prevent the accumulation of soil-borne pathogens specific to certain families of plant by alternating with an incompatible host [13,24]. However, although often a successful strategy, crop rotation is not always an economically viable strategy for farmers to adopt if the rotation crop is of low economic value. In addition to rotation, selective breeding programs aim to introduce plant disease resistance genes (for example R genes) into modern cultivars [25,26,27]. However, in some cases this can be challenging and there are several crop species for which are there are no resistant cultivars available [25]. In addition, pathogens can quickly overcome plant host resistance mechanisms, particularly when resistance is encoded for by a single gene [25]. As an example, rice cultivars that are resistant to *M. oryzae* typically become ineffective every 2–3 years [18]. These problems combined have led to the search for further alternatives. Increasingly, it is being realised that the microorganisms living within soil and in close association with plant roots can make large contributions to plant health and could be engineered as biocontrol agents.

## 2. Plant-Microbe Interactions and Their Effect on Plant Health

The vast majority of eukaryotes, including plants, interact extensively with a diverse community of microorganisms. In plants, interactions particularly emerge at the interface between the plant roots and the soil environment, whereby bacteria from the soil abundantly colonise the soil layer, known as the “rhizosphere”, that is immediately surrounding and influenced by the plant root system [28,29,30,31]. Several microbial species are also capable of attaching to the root surface (a region called “the rhizoplane”) and a small subset of the soil community additionally enter the plant root tissue [29,30,32]. The latter group of microorganisms are adapted to survive within the inter or intracellular spaces within the plant roots, which are collectively known as the “endophytic compartment” [29,30,32]. Advances in next generation sequencing (NGS) techniques have facilitated deeper probing into the microbial ecology of the plant root microbiome. Although abiotic factors such as soil characteristics appear to influence the composition of the microbiome, it is also clear that host genetics play a key role in root microbiome assembly and plants are likely to select beneficial species from their environment [33,34,35,36]. Factors such as differences in root architecture can influence this assembly and selection process, for example by influencing soil characteristics, as well as the organisation and structure of root cells [32,37,38]. Additionally, around 20–40 % of photosynthetically fixed carbon is exuded from plants into the rhizosphere; these exudates include a broad range of organic compounds that can be utilized by microorganisms and may help to select certain species from the soil community [28,32,39,40].

It has been known for some time that both soil and plant-associated microbes can contribute to plant health, since the presence of certain microbial species can result in a reduction in plant disease incidence and severity [41,42,43,44]. Additionally, specific isolates from the plant root microbiome produce a range of secondary metabolites that can inhibit plant pathogens both in vitro and in vivo [15,18,20,42]. In particular, the potential of a Gram positive genus of Actinobacteria, called *Streptomyces*, has drawn the attention of many in the scientific and industrial communities. Streptomycetes are saprotrophic organisms, best known for their role as producers of clinically useful antibiotics, of which they are responsible for approximately 55% [45,46,47]. This genus is characterised by their polar filamentous growth, their spore-forming capabilities, and particularly, their extensive secondary metabolism [45,47,48]. These secondary metabolites are known to have a diverse range of activities and have been used for a wide range of applications, including as antibacterials, antifungals, anti-cancer and anti-helminthic drugs [45,47,49,50]. Since *Streptomyces* are abundant in soil and have been shown to suppress a range of phytopathogenic organisms both in vitro and in vivo, these organisms are gaining interest as potential biocontrol agents that could be used in place of conventional chemical treatments [20,51]. In this review, we specifically focus on reviewing research that investigates the role that *Streptomyces* can play in inhibiting pathogens of cereal crops, particularly fungal pathogenic species. We focus on this in particular, due to the global importance of cereal crops, the large socioeconomic impacts of yield losses caused by fungal disease and the lack of other alternatives for controlling many of these pathogens. Several excellent reviews [18,20,51,52] have discussed the general potential of *Streptomyces* as biocontrol agents or their application to one specific crop species and we extend this literature by specifically focusing on cereal crops.

## 3. *Streptomyces*—Plant Interactions

The evolution of the first true streptomycetes approximately 450 million years ago is thought to have been largely stimulated by the transition of plants onto land, approximately 550 million years ago [46]. Millions of years of plant-streptomycete interactions may explain why *Streptomyces* are often found to be abundant in the rhizosphere and roots of a variety of different plant species. For example, *Streptomyces* have been shown to be enriched in the roots and rhizosphere of *Arabidopsis thaliana* [34,35,53], as well as in important crop species, such as potatoes [54], rice [55], wheat [56,57] and oilseed rape [36]. A long period of coevolution with plants might also have resulted in several aspects of the growth and metabolism of this genus. For example, selective pressures to break down plant material are thought to have driven the evolution of a saprotrophic and filamentous lifestyle, which would have enabled early streptomycetes to penetrate living and dead plant material in order to access otherwise unavailable nutrients stored in complex molecules, such as cellulose [46,58]. This may have eventually led to an endophytic lifestyle, and indeed, fluorescent microscopy has shown that streptomycetes can exist endophytically within the roots of several different plant species, including lettuce, wheat and pea, and that they may be able to penetrate plant roots by entering openings that occur at the bases of root hairs and lateral roots [57,59,60,61]. *Streptomyces* are also capable of producing an array of cellulolytic and hydrolytic enzymes that might allow forced entry into plant material by breaking down the epidermal cell walls and middle lamellae between plant cells [20]. Their ability to produce a diverse array of antimicrobial secondary metabolites may additionally allow them to compete for niche space and the carbon-rich resources that are exuded by plants.

Given their ability to colonise plant roots and produce potent antimicrobial secondary metabolites, the genus *Streptomyces* are becoming an increasingly obvious choice when looking for novel biocontrol agents (Table 1). This is particularly the case, as in addition to contributing to plant protection, members of this genus are frequently found to contribute to plant growth promotion (PGP) under both ambient and stressful environmental conditions, such as high salinity [20,46,62,63,64]; these additional benefits could form the basis for highly desirable biocontrol agents that can both enhance plant growth and protect against disease.

**Figure 1:**
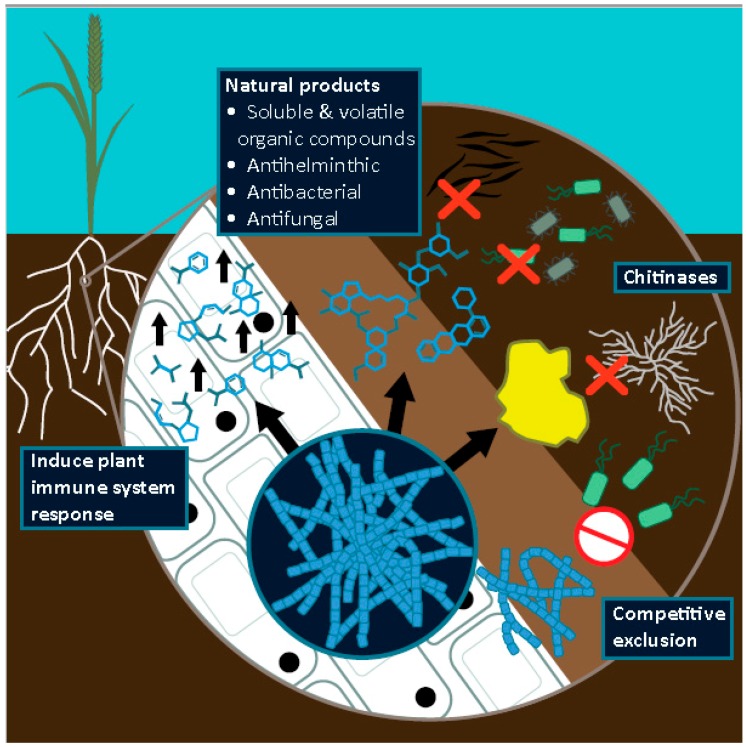
A summary of the major mechanisms by which *Streptomyces* biocontrol strains can protect cereal crops from disease.

It is important for us to note that, although many *Streptomyces* are either beneficial or passive colonisers of the plant microbiome, certain species have evolved a phytophathogenic lifestyle. Perhaps the most well-studied example is *Streptomyces scabei*, the causative agent of common potato scab [80,81,82]. Several virulence factors have been found to be associated with this disease-causing lifestyle, including small molecules such as coronafacic acid and thaxtomin, the latter of which is located on a pathogenicity island within the genome of plant-pathogenic strains [83]. Only a handful of *Streptomyces* species have these genes, and it is suggested that their acquisition was a singular event and does not represent the interactions that are characteristic of plant-*Streptomyces* relationships. Indeed, out of over 500 isolated *Streptomyces* species, only 10 are deemed to be pathogenic [20,84]. Thus, there is a huge diversity of strains that could be screened for their potential to act as beneficial biocontrol agents. In the following sections we review the multitude of ways in which *Streptomyces* species can contribute to the suppression of cereal crop diseases, both directly and indirectly (Figure 1). We also extend this to a discussion of how such strains might be applied to cereal crops in practice, and the factors that can influence the competitiveness and efficacy of biocontrol agents, and thus need to be considered during the development of such strains as biocontrol agents.

### 3.1. Streptomyces in Disease Suppressive Soils

*Streptomyces* can confer plant host protection against pathogens in the soil, rhizosphere and endosphere directly, through the production of antimicrobial compounds or via specific enzymes (Figure 1) [48]. Disease suppressive soils are perhaps some of the best known examples of microbial-based defense against soil-borne pathogens, and several studies have used these soils as a source of novel bioactive microbial strains [41,44]. Suppressive soils are those in which plants are protected from infection, due to the antagonistic activities of a community of microorganisms, or a specific microbial species, found in the soil and rhizosphere community [41]. Such soils often occur in areas in which there has been continuous monoculture and can be disrupted by particular farming practices, such as crop rotation [41,44,68]. The mechanisms underpinning suppressiveness are only just beginning to be understood, but antibiotic-producing *Streptomyces* species have often been found to be enriched in these soils; a combination of metagenomics, strain isolation, genome sequencing and genome mining has enabled the isolation of contributing species and their associated bioactive compounds [68,85,86,87,88]. For example, the strain *Streptomyces* S4-7 was originally isolated from a Korean soil that showed suppressiveness against *Fusarium* wilt disease [68]. Following genome sequencing, this strain was found to encode 35 biosynthetic gene clusters for producing putative antimicrobial agents. A novel thiopeptide was purified and shown to have potent inhibitory activity against fungal cell wall biogenesis in *Fusarium*, suggesting natural products such as this may be contributing to the disease suppressive nature of the original soil [68]. *Streptomyces* species were also found to make a major contribution to the suppressiveness of light coloured *Sphagnum* peat in Finland, which inhibits the development of a range of soil-borne pathogens, including *Rhizoctania solani* and *Fusarium* spp., and is therefore commonly adopted for glasshouse cultivation [41,89]. An analysis of the microbial composition of this soil led to the isolation of the bioactive strain *Streptomyces griseoviridis*; this was then used to formulate the broad-spectrum biofungicide Mycostop^®^, which is active against a number of crop diseases, including wheat head blight caused by *Fusarium* species [70].

### 3.2. Antimicrobials against Phytopathogens of Cereal Crops

In addition to disease suppressive soils, there have been many efforts to isolate strains of *Streptomyces* from other environments that are capable of inhibiting some of the most detrimental cereal crop pathogens. Many studies have found *Streptomyces* species that can inhibit a range of phytopathogens in vitro, including *Magnaporthe oryzae* (responsible for rice blast), *Gaeumannomyces graminis* var. *tritici* (the cause of wheat take-all fungus), *Fusarium* species (responsible for head blight, root rot, wilt and grain contamination in a variety of species), as well as *Rhizoctani solani* (a soil-borne pathogen with a wide host range) [8,15,18,66,71] (Table 1). However, such inquiries only form the beginning of a chain of experiments required to identify novel biocontrol agents. In soil, *Streptomyces* bacteria interact with a diverse community of both prokaryotic and eukaryotic organisms, which may alter their competitive ability and potential to produce antimicrobial compounds. Thus, there is a real need to demonstrate that isolates can also confer plant protection in vivo, both in greenhouse experiments and in field trials.

Several greenhouse and growth chamber experiments have been carried out with bioactive compounds purified from cultures of *Streptomyces* species (Table 1). For example, a soil isolate, named N2, was shown to inhibit a broad spectrum of phytophathogenic fungi in vitro, including the mycelial growth of *R. solani*, as well as the germination of its sclerotia [75,78]. Sclerotia mediate the dispersal, propagation and long-term survival of the fungus in soil and are persistent under unfavourable environmental conditions [75,78]. A novel antifungalmycin was found to be responsible for the inhibitory effects of N2 [78], and when directly applied, was also able to reduce the symptoms of sheath blight on rice leaves and in pot experiments [75]. Another study has shown that culture filtrates of the strain *Streptomyces* globisporus JK-1 can control *M. oryzae* more effectively than tricyclazole, a commonly used chemical fungicide for the control of rice blast fungus [65]. Indeed, several antifungal compounds purified from *Streptomyces* species have been commercialized as fungicides against *M. oryzae* infections, for example, Kasugamycin (isolated from *S. kasugaensis*) is commercially produced under the trade name Kasumin, and is used in Japan to protect against rice blast disease [18].

Other studies have used live strains of *Streptomyces* during in vivo trials rather than purified bioactive compounds [15,18,67,69] (Table 1). For example, the strain *Streptomyces* BN1, isolated from rice grains contaminated with *Fusarium*, was able to mitigate the reduction in seedling length caused by *Fusarium* when applied as a spore preparation to seeds. BN1 also significantly reduced *Fusarium* head blight symptoms when sprayed onto wheat heads [67], suggesting that the application of viable spores can be an effective way to reduce the competitive ability of pathogenic strains. Spore-coatings were also used in a study investigating the ability of *Streptomyces* species (isolated from healthy cereal crops) to inhibit wheat take-all infection by *G. graminis* var. *tritici* [15]. Spore-coated seeds significantly reduced wheat infection in field soils that were infested with the take-all fungus [15]; this may have been aided by the ability of these strains to colonise the endophytic compartment of wheat roots [57]. There is currently a lack of wheat cultivars with resistance to *G. graminis* and chemical agents are variable in their ability to control the disease [13,15,44]. *Pseudomonas* species have been investigated as potential biocontrol agents against take-all, but often these strains only colonise wheat plants during the early stages of growth before being out-competed, and they are also sensitive to desiccation [15,44]. *Streptomyces* may make a viable alternative, since their saprotrophic and spore-forming lifestyle means that they survive well under unfavourable conditions [48]. They can also colonise the mature roots of cereal crops [15].

### 3.3. Enzymatic Control of Phytopathogens: Chitinases

The majority of *Streptomyces* species encode an enormous variety of secreted proteins that have a diverse range of extracellular activities [90]. This includes the production of enzymes called chitinases, which degrade the biomolecule chitin (Figure 1). Chitin is an insoluble, nitrogen-containing polysaccharide that is abundant in fungal cell walls [90,91]. *Streptomyces* are unusual amongst bacterial taxa in that they can use it as both a carbon and a nitrogen source [90]. Chitinases isolated from *Streptomyces* species have been shown to inhibit a broad spectrum of phytophathogenic fungi and oomyctes in vitro, including economically important genera, such as *Fusarium*, *Rhizoctania* and *Pythium*, and are therefore receiving increasing interest from a biocontrol perspective [92,93,94,95]. Chitinases are thought to contribute to the in vivo antifungal activity demonstrated by the broad-spectrum biocontrol strain *Streptomyces lydicus* WYEC108, which is the active ingredient in the commercially-available biocontrol agent Actinovate^®^. Purified chitinase from this species was able to lyse the cell walls of various phytopathogenic fungi, including several species of *Pythium*, which can cause root rot in a variety of cereal crops [79]. Finally, transgenic expression of the *S. griseus* chitinase-encoding gene *chiC* conferred an increased level of resistance to the blast fungus *Magnaporthe grisea* on rice plants, suggesting that *Streptomyces* species may also represent an important genetic resource [96].

### 3.4. Direct Inhibition by Volatile Organic Compounds

In addition to soluble compounds and enzymes, many *Streptomyces* are prolific producers of Volatile Organic Compounds (VOCs) [97]. These are characteristically small compounds with low molecular weights and high vapour pressures, meaning that they can easily diffuse through water and gas-filled pores in soil [43,98]. Strains can produce complex and diverse mixtures of VOCs that have a diverse range of functions, many of which are only just beginning to be understood [99]. Several VOCs have been identified that have antimicrobial activities against phytopathogenic species (Figure 1), for example, profiling of *Streptomyces* strains isolated from a soil suppressive to *R. solani* revealed that a range of VOCs had potent antifungal activity against the pathogen in vitro and additionally resulted in an increased plant root and shoot growth [85,86,97]. Other studies have also isolated streptomycete VOCs active against *R. solani* in vitro, in addition to species of *Fusarium* and *Aspergillus* [72,77]. Such studies introduce the possibility that VOCs could be applied as biofumigants to suppress the growth of pathogenic species and may also have significant impacts on soil-borne pathogens when produced by *Streptomyces* species growing in the rhizosphere. However, more studies are needed to verify that these compounds are both produced in vivo in the plant root system and effective under natural conditions.

### 3.5. Antihelmintic Compounds

In addition to antimicrobials, *Streptomyces* are also known to produce potent anthelmintic compounds. This includes the compound avermectin, produced by *Streptomyces avermitilis*, which can cause extensive mortality to nematode populations in vivo [100,101]. Cereal cyst nematodes parasitise host plants by forming root cysts, in which they tap into the nutrients present in the plant vascular system; as a result they can cause extensive damage to wheat and maize crops and are prevalent in the majority of the cereal growing regions of the world [102,103]. A small number of studies have documented *Streptomyces* species that can control populations of cereal cyst nematodes [104,105,106]. Given the enormous variety of natural products produced by *Streptomyces* strains and the fact that, in soil, they are likely to encounter and compete with a diverse population of nematode species, a greater number of such compounds may be discovered.

### 3.6. Indirect Inhibition of Phytopathogens of Cereal Crops

In addition to direct inhibition via the production of antagonistic compounds, *Streptomyces* can also inhibit plant pathogens indirectly (Figure 1). The simplest way in which this can occur is via competitive exclusion, whereby strains take up niche space and resources, therefore preventing pathogens from colonizing [20,107]. This is not mutually exclusive from direct antagonism since antimicrobials may be produced as a byproduct of interference competition over the resources provided via plant root exudates or organic matter in the soil.

However, a further mechanism by which *Streptomyces* can indirectly provide protection to their plant host is though the activation of host resistance pathways (Figure 1) [20,108,109]. Several species of rhizobacteria are known to induce host defense pathways systemically, in distal parts of the plants, via a process known as induced systemic resistance (ISR) [20,108,109,110,111,112,113,114]. ISR results in an elevated and more efficient response to future pathogenic attack and induces several changes, including the accumulation of defence-related compounds, localised cell death and cell wall reinforcements [114]. Systemic acquired resistance (SAR) is another mechanism by which the plant immune system can become primed to rapidly respond to pathogenic infections, however this pathway is primarily activated by prior interactions with biotrophic phytopathogens and mediates the plant defense response via the upregulation of pathogenesis-related (PR) proteins [114].

Most rhizobacteria, such as species of *Pseudomonas* and *Bacillus*, are known to mediate ISR via the activation of phytohormone defense signaling pathways, including one or both of the jasmonic acid/ethylene (JA/ET) pathway or the salicylic acid (SA) dependent signaling pathway, but they do not induce the transcription of PR proteins involved in SAR [110,114]. In contrast, the induction of plant defense by *Streptomyces* species differs from traditional ISR, as it involves features of both ISR and SAR, as well as the cross-talk between many different phytohormone signaling pathways [110,111,112,113]. For example, RNA-sequencing experiments have demonstrated that the inoculation of oak trees (*Quercus robur*) with *Streptomyces* sp. AcH505 leads to the upregulation of wide variety of genes involved in both ISR and SAR, including those related to tryptophan, phenylalanine, and phenylpropanoid biosynthesis, in addition to PR genes and genes contributing to all of the JA, ET, SA and abscisic acid (ABA) signaling pathways [110]. Furthermore, the expression of these genes is only partially amplified by co-inoculation of the streptomycete with the causative fungal agent of powdery mildew (*Microsphaera alphitoides*) [110]. In this system, immune system priming by the streptomycete successfully results in the suppression of the fungus and also alleviates fitness costs associated with infection by preventing a reduction in the level of photosynthesis-related transcripts [110]. These results differ from experiments involving *Pseudomonas fluorescens*, which does not initially elicit a detectable defense-related response in *A. thaliana*; in these plants, defense signaling is only significantly elevated upon subsequent pathogenic infection [110,115]. In comparison, streptomycetes appear to activate plant defense response pathways even in the absence of a pathogen [110]. Similar observations have been observed for endophytic *Streptomyces* strains colonising *A. thaliana* [112,113]. These results suggest that, upon colonisation, streptomycetes may be recognised as mildly pathogenic by the host plant, resulting in the activation of defense-related pathways, including those related to SAR. However, since most streptomycetes lack pathogenic determinants, a full response is only elicited upon infection by phytopathogens [112].

The ability of streptomycetes to induce plant disease resistance may extend their potential as biocontrol agents, since strains that induce such a response may be highly effective at protecting their plant host against pathogenic infection in situ, even if they demonstrate poor bioactivity against phytopathogens in vitro via the production of antimicrobial secondary metabolites [110,113,116]. Indeed, the degree to which over 50 *Streptomyces* strains were able to inhibit the growth of *Phytophthora* species in vitro was shown to be a poor predicter of their ability to protect alfalfa and soybean seedlings in vivo [116]. Instead, the ability of strains to increase plant biomass upon inoculation was a stronger predictor of disease outcomes [116]. Thus, screening for biocontrol strains should not be limited to the results of in vitro bioactivity assays and better proxies that represent the ability of strains to elicit host defenses, and thus protect host plant species in vivo, should be developed.

## 4. The Potential of *Streptomyces* Bacteria as Efficient Biocontrol Agents

The ability of *Streptomyces* species to produce plant-protective compounds, such as enzymes, secondary metabolites and volatile organic compounds, as well as their ability to induce the plant immune system to rapidly respond to pathogens (Figure 1) suggests that they would be good candidates for biocontrol agents. Biocontrol strategies can overcome some of the issues of chemical pesticides by offering a low cost alternative with greater potential for long-term sustainability [117]. Since many of the strains being developed as biocontrol agents, such as *Pseudomonas* and *Streptomyces* species, are often naturally abundant in soils, it is likely that they will cause less damage to the surrounding ecosystem [20,84]. Additionally, microbes that have evolved in close symbiosis with eukaryotic organisms, such as plants, may cause fewer unwanted side-effects in other eukaryotic organisms, including humans [84,118]. One of the key issues of chemical pesticides is that disease-causing agents can rapidly evolve resistance. Streptomycetes have the advantage that apart from being a potentially co-evolving force that could engage in an arms race with pathogenic species, many also encode numerous putative antimicrobial biosynthetic gene clusters (BGCs), resulting in the simultaneous production of a multitude of different antibiotics with different modes of action; this could help to reduce the rate at which resistance evolves [48].

Currently, there are two commercially available biocontrol products whose active ingredients are live *Streptomyces* strains. They are Mycostop^®^ (*Streptomyces griseoviridis* K61 [70]) and Actinovate^®^ (*Streptomyces lydicus* WYEC 108 [119]). The strains are purchased as dried spore preparations and applied as a seed treatment, or as an irrigative growth medium additive. Both *Streptomyces* species have demonstrated PGP and disease suppressive characteristics in a laboratory setting [61,120]. However, their efficacy as disease suppressing agents in an agricultural scenario can be inconsistent. For example, Actinovate^®^ was found to be poor at supressing Fusarium Wilt disease (*Fusarium oxysporum* f. sp. *niveum*) of Watermelon in field trials [119], and whilst it promoted the growth of Summer Squash, it was inconsistent in its ability to provide protection against powdery mildew (*Podosphaera xanthii*) [121]. Another study that assessed the effectiveness of treating Barley (*Hordeum vulgare*) and spring wheat (*Triticum aestivum*) with Mycostop^®^ at the same field site over five years, showed that although there was an initial increase in yield in both crop species, the results were inconsistent across the years, with a similar inconsistency in disease suppression [122]. Despite Mycostop^®^ reducing the incidence of root rot overall, it performed poorly when compared to treatment with a conventionally used (although widely banned) organomercurial pesticide [122]. This study demonstrates that yearly abiotic variation as well as biotic variation between crop species can significantly impact the potential of biocontrol treatments. In addition, existing biocontrol strategies do not always match, or exceed, the performance of conventional pesticide treatments. The inconsistency of biocontrol strains such as Mycostop^®^ and Actinovate^®^ also demonstrates the need for a greater understanding of the factors that influence strain competitiveness and their long-term establishment within the root microbiome of different crop species.

**Figure 2:**
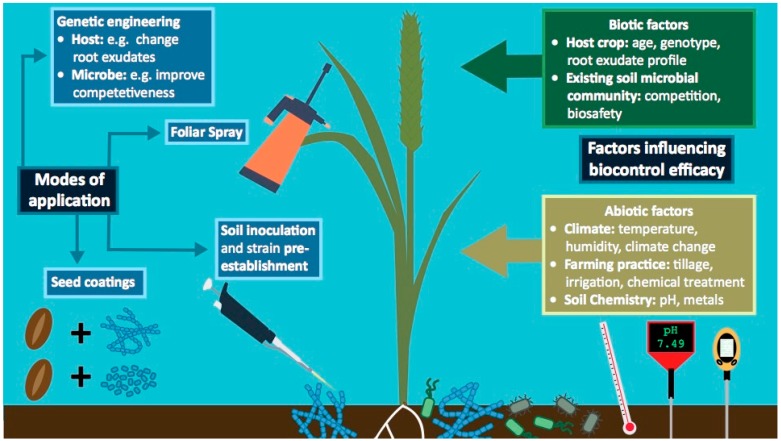
A summary of the major tools and methods available that could facilitate the application of biocontrol strains, as well as the major abiotic and biotic factors that can influence biocontrol efficacy in the field.

There are numerous factors influencing the composition of soil and root-associated microbial communities, and that in turn could influence the success of biocontrol strategies (Figure 2). Broadly, these factors can be divided into two categories (Figure 2). Firstly, abiotic factors, such as soil type (which is defined by characteristics such as nutrient levels, water content, pH and trace metals) [123,124], climate (and climate change) [125] and farming practice (e.g., irrigation, fertilisation, tillage and pre-cropping [126,127]) can all impact on microbial assemblages. Secondly, biotic factors include host crop species [36,55,128], host genetics [55,129], root exude profiles [129,130], plant age at the time of application [131,132], and competing microorganisms already present in the plant microbiome [133]. Additionally, many of these factors may vary significantly each growing season, adding an additional layer of complexity to the factors that influence root microbiome assembly. A detailed understanding of how these factors influence biocontrol success, and how to mitigate them, is a priority for the development of consistently effective biocontrol strategies. Progress is beginning to be made on this front, for example the Microbiome Stress project is an ambitious open access database collating and analysing 16S rRNA gene amplicon sequencing data [134]. The goal is to identify how bacterial communities respond to various environmental stressors, information which could be used to predict the efficacy of biocontrol strategies in different environmental conditions. This will be particularly important for developing robust biocontrol strategies in the face of climate change.

### 4.1. Abiotic Factors Influencing Biocontrol Efficacy

Numerous studies have experimented with strategies to improve the consistency and effectiveness of *Streptomyces* biocontrol agents by changing abiotic factors, such as the soil environment [135]. For example, an early study found that the application of wood chip-polyacrylamide medium (PAM) around the plant root significantly increased the ability of *Streptomyces lydicus* WYEC108 to protect potato crops from *Verticillium* wilt (caused by *Verticillium dahlia*) [136]. By pre-inoculating the PAM medium with *S. lydicus* WYEC108 spores, the strain was able to germinate and establish mycelia with reduced competition from the surrounding soil microbiota. Application of the pre-inoculated medium led to a reduced level of pathogen infection, as *V. dahlia* had to traverse the wood chip-PAM mixture colonised by antibiotic-producing *S. lydicus* before invading the plant [136]. Similarly, another study showed that pre-inoculating soil with *S. analatus* S07, a strain originally isolated from an *Heterodera filipjevi* nematode cyst, significantly reduced the infection of wheat roots with this nematode in a field trial [106]. In order to give the *Streptomyces* strain an advantage within the soil environment, an established pure culture was added to ground wheat grain; this was then incubated at the strains optimal temperature, before being applied to the soil in field plots [106]. The efficacy of disease control by *S. analatus* S07 was shown to match that of an established nematicide, avermectin, which is significant given the damage avermectin can cause to the wider ecosystem [106,137]. Such studies suggest that reducing abiotic stress on the biocontrol strain by helping it become pre-established in the soil can improve the efficacy of biocontrol strategies.

Apart from strain inoculation, a wide range of agricultural practices are thought to influence the composition and establishment of species within the plant root microbiome, including irrigation [126], tillage [127] and different cropping practices [138]. Agro-chemicals such as pesticides and fertilisers are also known to influence the composition and functioning of the plant root and soil microbiome, in ways that can help to protect against crop disease [139,140]. For example, ammonia fumigation has been shown to suppress *Fusarium* wilt disease in Banana (*Musa acuminate Cavendish*) and also leads to a shift in the composition of the microbial community in the surrounding soil, with a significant reduction in the abundance of *Fusarium* species [140]. Other studies have suggested that when organic fertiliser is applied in combination with biocontrol strains, the extent of disease suppression can be further enhanced. For example, suppression of the disease-causing bacterium *Ralstonia solanacearum* by *Streptomyces rochei* is significantly increased when applied in combination with organic fertiliser [141]. It is thought that adding a biocontrol strain to organic fertiliser prior to treatment generates a more favourable soil environment for the strain, with more nutrients available to support growth, increasing root colonisation and biocontrol efficacy [142]. This strategy is known as bio-organic fertiliser application and is widely reported as an effective method of enhancing disease suppression [139,143,144].

There are numerous other examples of chemical additives that are being trialed to augment disease suppression in agricultural systems. For example, the addition of chemical factors known to promote antibiotic production in *Pseudomonas* (e.g., glucose and zinc) have been shown to increase biocontrol efficacy [145]. This implies that factors known to increase antibiotic biosynthesis in *Streptomyces* (for example N-acetylglucosamine, rare earth metals, such as scandium or siderophores [146], and some plant phytohormones [48]) could, where practical, be used as an additive in streptomycete biocontrol formulations to maximise disease suppression. Conversely, some chemical additives have been demonstrated to be detrimental to the biocontrol efficacy of *Bacillus* species in vitro, for example pesticides that contain heavy metals such as copper and zinc, and a number of fungicides and herbicidal compounds [147]. Despite this observation, biocontrol strain *Streptomyces* sp. A6 was found to be highly tolerant to a number of commonly used fungicidal compounds, and simultaneous application of the strain with these fungicides resulted in more effective *Fusarium* wilt control in pigeon pea (*Cajanus cajan*) and a 50% lower dose of fungicide was needed for effective crop protection [148]. This demonstrates that combining chemical and biological pest control methods can increase biocontrol efficacy, while simultaneously decreasing the required dose of chemical pesticides. Whilst together these studies imply that farming practices could be optimised to maximise disease suppression, comprehensive research into this is still lacking. Such research is complex, as it is likely that the best approach will depend upon the pathogen of concern, as well as the relevant climatic and edaphic conditions.

### 4.2. Optimising Biocontrol Delivery Systems Involving Streptomyces

Various methods are available for delivering biocontrol strains to crops and could further influence the consistency of biocontrol strategies (Figure 2). Products such as Actinovate^®^ and Mycostop^®^ come as dried formulations containing spores and mycelia; these can either be suspended in liquid and sprayed onto crops (foliar spraying), folded into the soil prior to sowing (soil inoculation) or be used as a seed coating [149,150]. Foliar spraying approaches often seem attractive, particularly in developed countries where equipment for spraying is already available. However, microbial suspensions can damage or clog machinery by settling out of solution, and stresses caused by passage through spraying apparatus (such as heat stress or shearing forces) can decrease biocontrol strain viability [151]. Foliar spray is also typically used for microbial inoculants designed to counter foliar diseases [152], and so may be less apt for controlling root-diseases such as wheat take-all fungus. Soil inoculation is another recommended mode of application, typically used if biocontrol strains are particularly vulnerable to desiccation [152]. As discussed previously, methods such as bio-organic fertiliser application [139,141,143,144] and strain pre-establishment [106] can increase biocontrol success when using this method. Often however, these strategies will add to the expense and complexity of applying disease suppressive measures, and the strategies used to augment biocontrol success can have unknown or even conflicting effects by altering the soil chemistry and microbiome composition [127,140].

The direct inoculation of biocontrol strains onto plant roots circumvents issues of soil-survivability measures, as the strain does not pass through an environmental medium prior to root colonisation. Examples of this include methods that apply biocontrol agents directly onto the plants root, such as fluid drill inoculation and root transplant dip. Both methods allow biocontrol strains to colonise roots in a controlled scenario; for root dip, roots of plant seedlings are incubated in a liquid cell suspension before transfer to the field [152] and in fluid drill methods seeds are allowed to pre-germinate within a gel containing the biocontrol strain [152,153]. In some cases, root dip has been shown to increase root colonisation by streptomycetes compared to soil inoculation [59], and this method has successfully been used to apply strains that can protect crops from diseases such as *Fusarium* wilt [154]. However, pre-germinating plants and manually inoculating the roots is labour-intensive compared to purchasing pre-coated seeds and also requires large quantities of bacterial inoculum to be grown for this purpose [150]. Fluid drill methods have also been shown to increase colonisation of plant roots by inoculated bacterial strains and a limited number of studies show that this can result in efficient disease suppression [155,156]. However, there is little work investigating the ecological impact of fluid drill gel application.

As mentioned, plants can also be colonised by coating the seed in a formulation of biocontrol strain spores or cells. Seed coatings use a variety of methods to adhere biocontrol strains to the seed surface. For example, seeds can be immersed in a microbial suspension and dried before germination (bio-priming) [157], or a liquid cell suspension or an adhesive is used to coat the seed in bacterial cells (called film coating) [150]. Seed coating technologies can effectively deliver biocontrol strains directly to the soil immediately surrounding a germinating seed and the rhizosphere [150,152] and there are numerous examples where seed coating approaches have proven effective at suppressing disease in both field and laboratory experiments [157,158,159,160,161]. This includes numerous studies showing that seed coatings are an effective delivery method for *Streptomyces* biocontrol strains [59,162,163,164] to cereal crops such as maize [73] and wheat [74]. While seed treatment is an effective inoculation method, practical issues such as shelf-life and storage conditions remain problematic in many cases [150,165]. However, certain spore preparations of streptomycetes have been suggested to have a greater potential for long-term viability [166].

### 4.3. Exploiting Plant Recruitment Mechanisms to Improve Biocontrol Agents

In addition to enhancing the competitiveness of strains when applied to seeds and soil, it is possible that the mechanisms that enable plants to selectively recruit certain microbial species from the soil could be exploited to improve the efficacy of biocontrol strains (Figure 2) [135,167,168]. As mentioned, plants exude approximately 20–40% of photosynthetically fixed carbon out of their roots into the surrounding soil [28,169]. This exudate contains a whole range of compounds, including those with low molecular weights, such as ions, amino acids, sugars and phenolics, as well as high molecular weight compounds, such as mucilage, other polysaccharides and proteins [39,169,170,171,172]. The release of exudates into soil results in a large increase in microbial abundance and activity in the region of soil directly surrounding the roots; this is known as the “rhizosphere effect” and occurs because many microbes are attracted to the carbon-rich nutrients exuded from the roots [28,39]. However, exudates could also act as a filtering mechanism, enabling plants to selectively enrich for specific microbial species with particular metabolic capabilities [39]. This hypothesis is supported by experiments that have profiled the root exudates of *Arabidopsis thaliana* and found that certain groups of exudate compound correlate with the abundances of particular bacterial taxa [40,131,171]. For example, various phenolic compounds have been suggested to act as specific substrates or signaling molecules for particular microbial species, since they positively correlate with the abundances of specific genera, including *Streptomyces* bacteria [40,131,171,173]. Stable isotope probing experiments that track ^13^C isotopes from plant metabolites to bacterial RNA, DNA or proteins have also revealed that different microbial taxa are actively metabolising the root exudates of different plant host species, presumably due to differences in exudate composition [36,169,174]. In addition to host plant species, root exudation can also be altered by abiotic and biotic factors. For example, several studies on barley and *Arabidopsis* plants have indicated that root exudate profiles change in response to foliar and soil-borne pathogens, which in turn leads to changes in the rhizosphere and endosphere bacterial community composition [175,176,177].

In addition to changes in abundance, root exudates may also alter the functionality of the root microbiome to the benefit of the host plant by altering microbial gene expression [131]. Increasing amounts of phenolic-related compounds are exuded by *A. thaliana* roots at later developmental stages and these have been shown to correlate with an increased number of microbial transcripts related to antimicrobial production, including streptomycin produced by *Streptomyces* species, independent of changes to bacterial abundance [131]. These antagonistic molecules may be beneficial to the plant at later developmental stages as it could encourage the suppression of pathogenic species or priming of the plant immune system, providing the host with protection against infection at the flowering stage [131]. Several plant root exudate compounds have also been shown to modulate the production of antimicrobials by *Streptomyces* species in vitro, including the plant phytohormones, salicylic acid, jasmonic acid and indole-3 acetic acid (IAA) [178,179].

Correlations between root exudate composition, microbial community structure and microbiome functionality open the exciting opportunity to tap into these chemical interactions in a way that enables improvements to crop productivity and health. For example, it may be possible to engineer plants that produce certain types of root exudate, which in turn improve the colonisation potential and efficacy of beneficial species and biocontrol agents, such as *Streptomyces* species. Indeed mutant lines of *Arabidopsis* that have been engineered to have altered root exudation profiles have been shown to recruit different types of bacterial species, including greater numbers of beneficial plant-growth-promoting rhizobacteria [129,180,181]. Thus, it may be possible to introduce similar changes into cereal crops through breeding or genetic modification. However, there is still a huge knowledge gap regarding that compounds act as signals and nutrients for bacteria of interest. Such cues are only known in detail for a small number of plant-microbe symbioses, such as the role of flavonoids in legume- rhizobia interactions [172]. The vast majority of other systems are not so well-defined. Tools such as stable isotope probing [169,182], metabolomics [170], dual RNA sequencing [183,184] and imaging mass spectrometry [185,186,187] are beginning to shed light on these interactions and may enable a more detailed understanding of plant-microbe interactions in the future.

### 4.4. The Biosafety of Streptomycete-Based Biocontrol Agents

As mentioned, there are currently only two commercially available biocontrol products whose active ingredients are publicly listed as live *Streptomyces* strains. Difficulties in preparing commercial products that are suitable for large-scale application and long-term storage are partly responsible for this, in addition to the lack of consistency of strain activity in situ [188]. However, other hurdles also exist, for example it is additionally necessary to determine that strains do not cause clinical toxicity or persist in the agro-environment for long periods of time [149,188]. Establishing whether this is the case requires extensive screening that can hinder commercialisation. For many strains that have been identified as candidate biocontrol agents, the non-target effects of their application are not well-established [51,189,190].

One potential concern surrounding the use of antagonistic bacteria, such as *Streptomyces* species, as biocontrol agents is that the diversity of secondary metabolites that they produce could exhibit extensive non-target effects, including those that are detrimental to human health [190]. For example, in addition to targeting pathogenic species, several groups of secondary metabolites are also known to target key components of human cells, leading to serious toxic side-effects [190,191]. This is the case for many polyene macrolide antibiotics that inhibit fungal pathogens by increasing the permeability of fungal plasma membranes, but also have a high affinity to cholesterol, which is abundant in mammalian cells [191]. There are very few studies that investigate the degree to which antimicrobials are actually produced by biocontrol agents in situ in the soil [189,190]. Some studies suggest that antimicrobials may only be produced at very low concentrations and play a minor role in inhibiting pathogens in the plant root niche, whereas traits that enable strains to efficiently dominate the use of space and resources, or to activate the plant immune response, may be more important for excluding pathogens and reducing disease incidence [192,193]. Many gene clusters encoding secondary metabolites are also tightly regulated, and so it is possible that they are only activated as a result of direct interaction with particular pathogenic species [48,194,195]. This latter scenario would be beneficial, as it would provide a targeted means to suppress the growth of plant pathogens and may produce fewer unwanted side-effects, such as toxicity to humans and the widespread evolution of pathogen resistance, than a blanket application of purified antimicrobial molecules at high concentrations. However, further studies on the behaviour and mode of action of biocontrol agents in vivo, as well as the concentrations of secondary metabolites that they produce in the soil environment, would inform the development of methods that ensure any non-target effects of compounds with broad-spectrum activities are limited in situ.

In addition to considering the possible side effects of streptomycete biocontrol agents on human health, strains that inhibit the growth of plant pathogenic microorganisms could also act antagonistically towards members of the indigenous microbial population in soil; possible non-target effects include the exclusion of microbial species that are beneficial to host plant fitness and the disruption of key biogeochemical cycles [51,189]. For example, several antimicrobial-producing streptomycetes have been isolated that are known to inhibit the initiation of plant host symbioses with mycorrhizal fungi [196,197,198]. This includes *S. griseoviridis*, which is the active ingredient of the commercially available product Mycostop^®^ [196]. Mycorrhizal fungi play an important role in phosphorous cycling in soil as well as plant nutrient acquisition, and their inhibition can result in reduced plant health and biomass [196,197,198]. Similarly, several streptomycetes are known to inhibit nodule formation by nitrogen-fixing bacterial species in the roots of leguminous plants [51,199,200]. For example, the inoculation of *S. kanamyceticus* was found to reduce nodule formation by the nitrogen-fixing species *Bradyrhizobium japonicum* in soybean; antagonistic molecules were suggested to be responsible for this, since nodulation levels recovered when antibiotic resistant strains of *B. japonicum* were co-inoculated with the streptomycete [199]. Despite these findings, it should be noted that not all streptomycetes exert such inhibitory effects, and indeed, there are several contrasting studies showing that *Streptomyces* species can increase nodulation and mycorrhiza formation, whilst simultaneously suppressing pathogenic growth [61,197,198,200,201,202]. Therefore, negative side-effects are clearly strain-specific, meaning that careful selection and screening of candidate biocontrol species is required.

Although there are several studies demonstrating the influence of *Streptomyces* species on individual plant symbionts, such as rhizobia and mycorrhizal fungi, there are comparatively few studies investigating the effects of their application on the composition and functioning of the wider soil ecosystem and plant root microbiome. It is possible that when strains are inoculated at low abundances, such as via seed coatings, they have limited impacts upon the indigenous microbial community, particularly if the existing soil microbiome is highly diverse, as this can confer greater resilience to invading microbial species [192,203]. However, introducing strains in larger quantities, and to the extent that they out-number their normal population sizes in soil, could cause greater disruptions to the existing soil community [189]. Indeed, a small number of studies have demonstrated that the addition of streptomycetes and other biocontrol agents to soil can cause short-term alterations to the composition and diversity of the indigenous soil bacterial community [189,204,205,206]. Results of such studies are, however, highly variable and depend upon the biocontrol strain, inoculation technique, plant host species and soil conditions [189]. Additionally, any potential impacts of inoculants can be difficult to differentiate from those caused by natural shifts due to plant growth stage, seasonal changes and agricultural practices [189,207]. Furthermore, few studies investigate whether non-target effects persist beyond the period immediately following biocontrol strain application, or whether the microbiome recovers to its pre-inoculated state after a short period of time [189]. The potential for protective microbial inoculants to select for more virulent pathogenic strains over long periods has also been underexplored [51,208]. In the future, there is a crucial need for carefully designed experiments that investigate whether the application of antagonistic biocontrol strains can have a long-term influence on pathogen populations, the functionality of the soil microbiome and key environmental processes [189]. Studies could be informed by monitoring the abundance of important indicator species, such as mycorrhiza, which contribute to plant health and nutrient cycling [189], or the expression of indicative functional genes that are related to plant-beneficial processes, including enzymes involved in PGP such as 1-aminocyclopropane-1-carboxylate (ACC) deaminase [209], or mycorrhizal-inducible phosphate transporters, which have been identified in several different cereal plants [210,211]. Careful controls will also be needed to differentiate between the influence of antagonistic molecules produced by inoculated streptomycete species on the soil microbiome, versus other factors such as biocontrol formulation components and abiotic changes [189].

## 5. Conclusions and Perspectives

In summary, the use of microorganisms to suppress plant disease and increase crop productivity is gaining increasing interest as a sustainable alternative to chemical approaches to suppress crop disease. *Streptomyces* species have a long history of coevolution with plants and other organisms, and as a result, have evolved a plethora of secondary metabolites and enzymes that function to interact with host organisms and inhibit competitors. Many of these molecules can provide significant benefits to plants by promoting plant growth and reducing the incidence of plant disease. These characteristics, along with the resilience of this genus to environmental stressors, suggests that they could be extremely useful as biocontrol agents. However, as highlighted in this review, a highly complex, interconnected network of factors can influence the efficacy of biocontrol in the field. Research into these factors is lacking but should be made a priority in order to enable the wide-spread application of highly effective biocontrol agents to cereal crops globally. Optimising the mode of delivery of biocontrol strains, for example by decreasing abiotic and biotic stressors, has shown some success in assisting soil and root establishment by these strains and for increasing the potency of biocontrol. However, other factors that affect plant microbiome establishment, such as agricultural practices, remain less well-studied, despite the fact that biocontrol optimisation is likely to be farm-specific. It is possible that we may be able to exploit pre-existing signals between plants and microbes to increase the colonisation potential of desirable strains, but in most cases these specific signals remain to be identified. Additionally, in the vast majority of cases, the influence of candidate biocontrol strains on the indigenous microbial population, as well as key ecosystem functions and pathogen virulence has not been investigated; such studies will be crucial for enabling the wide-spread application of biocontrol strains that have limited non-target effects. For the future development of more consistent biocontrol strategies, the most successful approach is likely to be combinatorial, considering delivery mechanisms, formulation additives, agricultural practices and the specific details of plant-microbe interactions.

## Figures and Tables

**Table 1:** Economically important cereal crop pathogens and associated biocontrol studies involving *Streptomyces* species.

Pathogen	Cereal Crop Host	Symptoms	Impact	*Streptomyces* as Biocontrol
*Magnaporthe oryzae* (Rice blast)	Rice, Wheat	Panicle, leaf and head blast	Yield losses and mycotoxin contamination	Greenhouse and in vitro studies [18,65,66]
*Fusarium* spp.	All cereals	Head, root, crown and stem blight in addition to wilt and grain contamination	Yield losses and mycotoxin contamination	Greenhouse, in vitro and field studies [67,68,69,70,71,72,73,74]
*Rhizoctonia solani*	All cereals	Seedling damping off, and infection of stems, roots and foliage	Yield losses and reduction in grain quality	In vitro and growth chamber studies [64,66,71,75,76,77,78]
*Gaumannomyces graminis* (Wheat Take-all)	Wheat, Barley, Rye, Rice, Oat, Maize	Root lesions and rot that spreads upwards to aerial parts of the plant	Yield losses	In vitro and greenhouse studies [15,57]
*Pythium* spp.	Wheat, Barley, Rice, Maize	Seed damping off, as well as root and stem rot	Yield losses	In vitro and growth chamber studies [76,79]
